# An Assessment of the Knowledge, Attitude, and Practice of Probiotics and Prebiotics among the Population of the United Arab Emirates

**DOI:** 10.3390/foods13142219

**Published:** 2024-07-15

**Authors:** Turfa K. Alqaydi, Alaa S. Bedir, Abdelghafar M. Abu-Elsaoud, Khaled A. El-Tarabily, Seham M. Al Raish

**Affiliations:** 1 Department of Biology, College of Science, United Arab Emirates University, Al Ain 15551, United Arab Emirates; 201801251@uaeu.ac.ae (T.K.A.); ktarabily@uaeu.ac.ae (K.A.E.-T.); 2 Department of Nutrition, College of Medicine and Health Science, United Arab Emirates University, Al Ain 15551, United Arab Emirates; 201950078@uaeu.ac.ae; 3 Department of Botany and Microbiology, Faculty of Science, Suez Canal University, Ismailia 41522, Egypt; abuelsaoud@science.suez.edu.eg; 4 Department of Biology, College of Science, Imam Mohammad Ibn Saud Islamic University (IMSIU), Riyadh 11623, Saudi Arabia

**Keywords:** consumer behavior, dietary habits, dietary supplements, functional foods, gut health, health awareness, nutritional knowledge, population study, public health

## Abstract

Probiotics and prebiotics offer a range of advantageous effects on human health. The knowledge, attitudes, and practices (KAP) of individuals can impact their inclination to consume probiotics and prebiotics. The main objective of this study was to examine the KAP of the people in the United Arab Emirates (UAE) about probiotics and prebiotics consumption. Additionally, the study aimed to assess the impact of KAP and sociodemographic factors on the use of probiotics and prebiotics. In order to accomplish this objective, a verified online questionnaire was used with a five-point Likert scale and distributed using an online platform (Google Forms). A cross-sectional research, non-probability sampling was implemented, and G*Power statistical power analysis was used to estimate a sample size of 385 participants. A total of 408 replies were gathered. The population under study consisted of residents in the UAE between the ages of 18 to 64 years old, excluding populations under the age of 18 and those living outside the UAE. A total of 392 participants met the criteria for inclusion in this study. The research ethics committees of UAE University granted the study approval (ERSC_2024_4359), and the validity of the findings was confirmed through face-to-face interviews with around 50 individuals and a Cronbach’s alpha test with result of 0.84. The statistical software SPSS version 29.0 for Mac OS was utilized to examine the relationships between KAP variables, including Chi-square tests and Pearson’s correlation coefficients. The tests were selected based on their capacity to handle categorical and continuous data, respectively. The female population was 85.2% of the total, while the male population accounts for 14.8%. The age distribution of participants shows that the largest proportion, 68.4%, falls within the 18–24 age range. Out of the participants, 61.5% held a bachelor’s degree. Most of the participants, 56.4%, were students, while 29.1% were employees. The average results indicate a significant inclination towards probiotics and prebiotics, as demonstrated by the scores above the midpoint for the six knowledge questions (M = 2.70), six attitude questions (M = 3.10), and six practice questions (M = 3.04). Several studies have examined this phenomenon; however, additional research comparing individuals in the UAE is necessary to fully comprehend the influence of KAP on the consumption of probiotics and prebiotics in the UAE.

## 1. Introduction

The recent shifts in people’s lifestyles, characterised by increased sedentary behaviour, have resulted in a decline in physical activity [1]. This can be attributed to a dependence on cars and delivery services, advancements in technology that minimize physical exertion, and changes in living standards and habits [1,2]. Consequently, these lifestyle changes have had a significant impact on overall health. Furthermore, the increase in population has stimulated farmers and scientists to achieve notable advancements in food production [2]. However, advancements require the use of chemical fertilizers and pesticides to alleviate plant and animal diseases and improve plant growth [3]. This could potentially impact human health and alter nutritional compositions. As a result, many people are currently looking for ways to enhance their well-being and regain balance in their bodies [2,3]. An illustration of this is the gut microbiota [1,2]. 

Healthcare professionals have highlighted the vital importance of gut microbiota in the functioning of the human body [1,2,3]. There is a belief that changes in living conditions, particularly those focused on enhancing hygiene, are leading to changes in factors that influence the development of intestinal microbiota. Moreover, the administration of antibiotics, particularly in children, disrupts the balance of microorganisms in the gastrointestinal tract [3]. Usually, people look for natural remedies to cure themselves, especially as there has been an increase in diseases that can be managed or avoided using natural methods, such as cardiovascular disease, obesity, and type 2 diabetes. A widely recognized treatment for these conditions involves the utilization of prebiotics and probiotics [4,5].

Probiotics are “live microorganisms which, when administered in adequate amounts, confer a health benefit on the host” [6,7]. Probiotic strains are present in a wide range of food products that are continually growing [7]. Probiotics can be found in different forms in the market, including functional food and beverages such as dairy products, non-dairy beverages, breakfast cereal, baked foods, fermented foods that are made through desired microbial growth and enzymatic conversions of food components (for example, fermented meat products), and dry-food probiotics [8]. They are also found in dietary supplements (including food supplements and nutritional supplements), speciality nutrients (such as infant formulations), pharmaceutical preparations, and animal feed [8,9]. The limited level of awareness and adoption of probiotics and functional foods among consumers presents a clear market obstacle [7,9]. 

Probiotics consist of various strains of the following genera: *Lactobacillus*, *Escherichia coli*, *Bifidobacterium*, *Enterococcus*, *Saccharomyces*, *Pediococcus*, *Lactobacillaceae*, *Streptococcus*, and *Leuconostoc*, which have been utilized to obtain defined health benefits [1,9,10]. The two most popular microorganisms used as probiotics are *Bifidobacterium* spp. and lactic acid bacteria (LAB). There are many species that contain probiotic strains, for example: *Lactobacillus acidophilus*, *Lactiplantibacillus plantarum*, *Lacticaseibacillus casei*, *Ligilactobacillus salivarius*, *Limosilactobacillus reuteri*, *Lacticaseibacillus rhamnosus*, *Lactobacillus delbrueckii* subsp. *Bulgaricus*, and *Pediococcus pentosaceus* [10,11], as well as strains from other bacterial species: *Lactococcus lactis*, *Leuconostoc*, *Leuconostoc mesenteroides*, *Bacillus subtilis*, *Weizmannia coagulans*, *Enterococcus faecium,* and *Bifidobacterium* spp. [10,11]. 

Some strains of *Bifidobacterium* spp. produce bacteriocins known as bifidocins, which possess antimicrobial activity against bacteria, including *E. coli*, *Listeria monocytogenes*, and *Staphylococcus aureus*, as well as some yeasts, *Saccharomyces cerevisiae* var. *boulardii* [12,13]. These genera are highly effective in enhancing cognition by regulating oxidative stress, apoptosis, and inflammation [12]. Furthermore, they play a role in controlling immune responses and have historically been employed to combat infections and inhibit the growth of pathogens [13]. Moreover, they are regarded as a promising treatment modality for potentially managing neurodegenerative disorders [14]. 

Probiotics exert many different beneficial effects via various gut microbiota–organ axes, such as gut–liver, gut–oral, gut-brain, gut–skin, gut–lungs, and gut–bone [15]. Strains of *B. longum*, *B. breve*, *B. infantis*, *L. helveticus*, *L. rhamnosus, L. plantarum*, *L. casei*, *L. acidophilus*, and *Bifidobacterium* can help improve oral health, intestinal health, immunity, nutritional status, stress response, and cognitive abilities; regenerate skin; and prevent obesity, osteoporosis, and psychiatric disorders like anxiety, depression, and mood disorders [14,15].

Gut–liver interactions maintain the balance of intestinal microorganisms and promote liver health, preventing the development of liver diseases [15]. Meanwhile, gut–lung interactions enhance mucosal immunity and combat bacterial and allergic infections, maintaining the health of the intestinal and respiratory systems [16]. It was discovered that a duration of four weeks of consumption is sufficient to produce quantifiable outcomes [11,17,18]. In addition, probiotic supplementation has a beneficial impact by decreasing the permeability of the intestines and the presence of lipopolysaccharides in the bloodstream while also increasing the concentrations of tight junction proteins [17,18]. Thus, clinical studies demonstrated decreases in serum lipopolysaccharide levels [18,19]. 

Probiotics are regarded as supplementary treatments to conventional therapy, alongside vitamins, minerals, and other dietary supplements [19]. The association of probiotics with well-being has a long history [9]. Prebiotics are a substrate that is selectively utilized by host microorganisms, conferring a health benefit [20]. This leads to specific changes in the composition and/or activity of the gastrointestinal microflora, resulting in improved well-being and enhanced health for the host [19,20]. An example of prebiotics is fibers, which are the edible parts of plants or analogous carbohydrates that are resistant to digestion and absorption in the human small intestine [21], such as inulin and fructooligosaccharides (FSO). They undergo fermentation in the intestine, promoting the growth of beneficial bacteria already present in the gut [6,22,23,24]. 

Prebiotics are classified according to specific criteria. First, the compound must be able to endure the stomach’s acidic conditions and resist degradation by enzymes found in mammals. Furthermore, it is imperative that the substance is not absorbed by the gastrointestinal tract and is easily fermentable by gut bacteria [24]. Finally, it should specifically stimulate the proliferation of advantageous bacteria to improve overall well-being [25]. 

Fructooligosaccharides (FOS), galactooligosaccharides (GOS), and inulin are types of dietary fibers that are present in various compounds [26,27]. They have been associated with neurological well-being and promote the production of advantageous short-chain fatty acids that improve glucose tolerance, increase the number of enteroendocrine cells (EECs), release peptides involved in fat removal, enhance intestinal permeability, and decrease harmful fermentation products that positively affect cardiovascular risk factors in obese individuals [28,29]. Consequently, they bolster immune responses in the gastrointestinal tract and potentially aid in maintaining a harmonious microbiota [30,31,32]. 

Furthermore, prebiotics contribute to the improvement of gut health by reducing lipopolysaccharide levels [1,30]. As a result, this process improves the strength and reliability of the intestinal barrier and decreases inflammation linked to obesity and metabolic disorders [31]. Moreover, it is essential to decompose substances that cannot be digested, such as dietary fibers, which produce short-chain fatty acids (SCFAs) such as acetic acid, propanoic acid, and butyric acid [32]. The association between SCFAs and reduced obesity and enhanced insulin sensitivity has been established [33,34]. 

Prebiotics are present in various food and beverage products. For example, unprocessed turmeric contains curcuminoids, arabica coffee is rich in compounds such as chlorogenic acids and polyphenols, and chicory root coffee is a caffeine-free alternative that contains inulin, guar gum, and acacia gum [32,34,35]. These substances are readily metabolized in the gastrointestinal tract and can also be present in orange juice and non-bovine milk [36]. Prebiotics can potentially improve nutrient uptake, thereby facilitating the proliferation of beneficial bacteria in the gastrointestinal tract [23,35,36,37,38].

Recent research has examined the effects of probiotics and prebiotics on alleviating various health conditions, such as mental health issues, particularly those managing irritable bowel syndrome (IBS), enhanced blood sugar regulation, gastrointestinal transit time, and ulcerative colitis [39,40].

Utilizing knowledge, attitudes, and practices (KAP) research techniques provides insight into individuals’ beliefs, thoughts, and behaviours regarding specific subjects [41,42,43], aiding in comprehending the utilization and avoidance of probiotics and prebiotics [44,45]. For example, a study evaluated health science undergraduates’ knowledge, attitudes, and practices regarding probiotics for the gut–skin axis [46]. The study revealed significant differences in the understanding and usage of probiotics. Significantly, a considerable 48.0% of health science undergraduates displayed commendable knowledge, whereas 67.4% maintained a neutral attitude and 69.2% showcased inadequate practices [46]. 

Similarly, a study was conducted in Romania to evaluate consumer awareness, knowledge, and interest in prebiotics, uncovering discrepancies in the participants’ knowledge levels [24]. A significant correlation was observed between age and knowledge levels, indicating that younger participants exhibited greater awareness and comprehension of prebiotics compared to older participants (*p* < 0.05) [24]. In contrast, the results from Saudi Arabia revealed substantial disparities in knowledge levels between gastroenterologist (GI) physicians and dietitians in Jeddah [45]. According to Eid et al. [45], 76.2% of GI physicians showed greater knowledge compared to 52.4% of dietitians. However, only 83.3% of GI physicians believed in the advantages of probiotics and prebiotics for patients with IBS, while only 50.0% of dietitians shared this belief [45]. Eid et al. [45] also conducted in Riyadh, Saudi Arabia, found that 56.3% of health science students had moderate levels of knowledge based on their KAP scores. The study also identified significant differences in knowledge, practice scores, and attitude scores based on various demographic factors [45]. 

In addition, another study by Radwan et al. [47] offered valuable information regarding the utilization of dietary supplements for COVID-19 patients among the population of the United Arab Emirates (UAE). A significant prevalence of diverse supplements was discovered, with special foods being the most prevalent (95.5%). Curiously, healthcare practitioners did not serve as the main source of information [47]. Instead, social media (40.4%) and personal networks (28.7%) played significant roles, as reported by Radwan et al. [47]. The study also found that demographic factors such as gender, age (over 40 years), and Asian ethnicity were associated with increased use of supplements and dependence on healthcare professionals [47]. 

Another study by Barqawi et al. [48] investigated the KAP of the population in the UAE regarding the microbiota and its composition [48]. The results obtained from the study involving 419 participants indicated that there is a lack of comprehensive knowledge regarding the microbiota, as only 29.3% of the participants demonstrated a satisfactory level of understanding. Significantly, being a university student and a healthcare professional were strong indicators of knowledge about the microbiota [48]. In addition, although there was awareness of probiotics, only 9.1% of participants demonstrated a high level of knowledge [48]. Furthermore, a significant percentage of individuals without medical backgrounds (42.4%) and healthcare professionals (34.6%) indicated that they had used antibiotics without obtaining a prescription [48]. 

Communicating the health benefits of functional food products to the public is essential for improving public health [9,49]. The lack of research on prebiotics and probiotics, including KAP, in the UAE highlights the need for more scholarly investigation to address this deficiency. 

The primary objective of the current study is to examine the knowledge, attitudes, and practices related to the consumption of prebiotics and probiotics among the UAE population aged 18 years and above. Furthermore, the study aimed to evaluate the influence of knowledge, attitudes, practices and sociodemographic factors on the utilization of probiotics and prebiotics.

## 2. Materials and Methods

### 2.1. Research Design and Data Collection

The current study employed a cross-sectional survey design to investigate the knowledge, attitudes, and practices regarding prebiotics and probiotics among UAE residents aged 18 years and older. The survey instrument was disseminated to participants through digital platforms such as WhatsApp links or email. This approach aimed to facilitate the participation of diverse individuals, including students and other residents across various demographics within the UAE.

Data for the present study were collected using a structured questionnaire adapted from prior research [19,45,50,51]. The questionnaire, comprising 18 questions, was divided into four sections: sociodemographics, knowledge (Table 1), attitudes (Table 2), and practices (Table 3), and all of them consisted of six different questions. Each section included items measured on a five-point Likert scale ranging from 1 to 5. The questionnaire was administered to diverse participants to ensure a comprehensive representation of perspectives on probiotics and prebiotics. 

The overall Cronbach’s alpha coefficient for the KAP questions was 0.838, indicating acceptable internal consistency and reliability of the study instrument. The research adhered to ethical standards, ensuring the confidentiality and anonymity of the participants. Informed consent was obtained from all respondents, who were assured of their right to withdraw from the study at any point. Before conducting the research, ethical approval by the UAEU Social Sciences Ethics Committee (Research Number: ERSC_2024_4359) was obtained on 13 March 2024.

**Table 1:** Knowledge questions.

NO.	Knowledge Question	Answer	Reference
KQ1	How is your knowledge about probiotics?	(No knowledge, Little knowledge, Neutral (undecided), Medium knowledge, High knowledge)	[19]
KQ2	How is your knowledge about prebiotics?	(No knowledge, Little knowledge, Neutral (undecided), Medium knowledge, High knowledge)	[19]
KQ3	Knowledge of probiotic and prebiotic health benefits?	(No knowledge, Little knowledge, Neutral (undecided), Medium knowledge, High knowledge)	[45]
KQ4	Knowledge of prebiotic types?	(No knowledge, Little knowledge, Neutral (undecided), Medium knowledge, High knowledge)	[45]
KQ5	What do you think are the constituents of probiotics?	(Live microorganisms, Synthetic drugs, Natural plant products, Chemicals in food, None)	[50]
KQ6	Are you aware of prebiotics, and how do they differ from probiotics?	(No aware, Little aware, Neutral (undecided), Medium aware, High aware)	[50]

KQ = knowledge question.

**Table 2:** Attitude questions.

NO.	Attitude Question	Answer	Reference
AQ1	Probiotic foods on the table represent health?	(Strongly disagree, Disagree, Neutral (undecided), Agree, Strongly agree)	[51]
AQ2	Probiotics cause decreased absorption of essential minerals?	(Strongly disagree, Disagree, Neutral (undecided), Agree, Strongly agree)	[51]
AQ3	Products containing prebiotics are not easily digested?	(Strongly disagree, Disagree, Neutral (undecided), Agree, Strongly agree)	[51]
AQ4	Prebiotic foods on the table represent health?	(Strongly disagree, Disagree, Neutral (undecided), Agree, Strongly agree)	[51]
AQ5	Prebiotics cause decreased absorption of essential minerals?	(Strongly disagree, Disagree, Neutral (undecided), Agree, Strongly agree)	[51]
AQ6	Products containing prebiotics are not easily digested?	(Strongly disagree, Disagree, Neutral (undecided), Agree, Strongly agree)	[51]

AQ = attitude question.

**Table 3:** Practice questions.

NO.	Practice Question	Answer	Reference
PQ1	I believe that probiotics are safe.	(Strongly disagree, Disagree, Neutral (undecided), Agree, Strongly agree)	[45]
PQ2	I believe that probiotics are effective in the treatment of gastrointestinal-related illnesses or symptoms.	(Strongly disagree, Disagree, Neutral (undecided), Agree, Strongly agree)	[45]
PQ3	Do you consume food products with probiotics?	(Never, Rarely, Occasionally, Frequently, Always)	[50]
PQ4	I believe that prebiotics are safe.	(Strongly disagree, Disagree, Neutral (undecided), Agree, Strongly agree)	[45]
PQ5	I believe that prebiotics are effective in the treatment of certain gastrointestinal-related illnesses or symptoms	(Strongly disagree, Disagree, Neutral (undecided), Agree, Strongly agree)	[45]
PQ6	Do you consume food products with prebiotics?	(Never, Rarely, Occasionally, Frequently, Always)	[50]

PQ = practice question.

### 2.2. Sample and Population

To ensure a broad representation of views on probiotics and prebiotics, the inclusion criteria for participants include volunteering and living in the UAE. In addition, participants were between 18 and 80 years old. Exclusion criteria include participants less than 18 years old and visitors to the UAE. Acceptable internal consistency and reliability of the study instrument were confirmed. 

Before conducting the online survey, face-to-face validation for around 50 participants was applied to check the validity of the questionnaire. Three hundred eighty-five individuals from various cultural groups and backgrounds were selected for the study. A sufficient sample size was chosen to ensure statistical validity. G*Power version 3.1.9.6 [52] was used to calculate the UAE population sample size. The sample size was determined based on an assumed population of 9.9 million UAE locals and expats [53]. For the equation, we used 95% as the level of certainty with which to determine the real population value, which means that there is a 95% likelihood that the real value of the estimate is inside the confidence interval.

### 2.3. Research Hypotheses and Questions

The study aimed to test the following research questions and hypotheses:

RQ 1: What is the knowledge, attitude, and practice level of probiotics and prebiotics in the UAE?

RQ 2: Are there any correlations between knowledge, attitudes, and practices concerning probiotics and prebiotics in the UAE?

RQ 3: Are there any correlations between knowledge, attitudes, and practices concerning probiotics and prebiotics in the UAE and sociodemographic data?

**H0:**  *There is no impact of knowledge, attitude, practices, or sociodemographic data on prebiotics and probiotics in the UAE*.

**H1:**  *There is an impact of knowledge, attitude, practices, and sociodemographic data on prebiotics and probiotics in the UAE*.

### 2.4. Data Analysis Tools

The data underwent analysis utilizing IBM-SPSS version 29.0 for Mac OS and Microsoft Office Excel 365. These software tools were selected for their robust statistical functionalities and user-friendly interfaces, enabling streamlined and precise data analysis. The reliability and internal consistency of the questionnaire were performed using Cronbach’s alpha reliability at the 0.05 level. The data normality was checked using both Kolmogorov–Smirnov and Shapiro–Wilk normality testing at the 0.05 level. 

Non-parametric data were described statistically in terms of frequency (*n*, %), mean, median, mode, and quartiles. The difference between scores was checked statistically using the Chi-square test. The interaction between study variables was determined using Pearson’s correlation and regression analysis at the 0.05 level. A blue–red heatmap was generated based on Pearson’s correlation coefficient using PAST statistical software version 4.04 (University of Oslo, Oslo, Norway).

## 3. Results

### 3.1. Questionnaire Reliability and Internal Consistency

The Cronbach’s alpha reliability coefficients for the scales knowledge (6 items), attitudes (6 items), and practices (6 items) were 0.809, 0.808, and 0.728, respectively, indicating high internal consistency within each scale (Table 4).

The overall reliability for the combined 18 items was also high at 0.840, and these results suggest that the scales are reliable tools for measuring the constructs of knowledge, attitudes, and practices, offering credibility to any findings derived from their use (Table 4). This high level of consistency across all scales underscores their suitability for further research and practical applications where these constructs are essential.

The Kolmogorov–Smirnov test revealed significant deviations from normality for the constructs of knowledge (D (392) = 0.068, *p* < 0.001), attitudes (D (392) = 0.133, *p* < 0.001), and practices (D (392) = 0.069, *p* < 0.001). Consistently, the Shapiro–Wilk test also indicated significant deviations from normality for knowledge (W (392) = 0.981, *p* = 0.001), attitudes (W (392) = 0.965, *p* < 0.001), and practices (W (392) = 0.987, *p* = 0.001) (Table 4). 

These findings suggest that the distributions of responses for knowledge, attitudes, and practices significantly differ from a normal distribution, underscoring the importance of selecting appropriate statistical methods for further data analysis i.e., for nonparametric data.

### 3.2. Sociodemographic Variables

**Figure 1:**
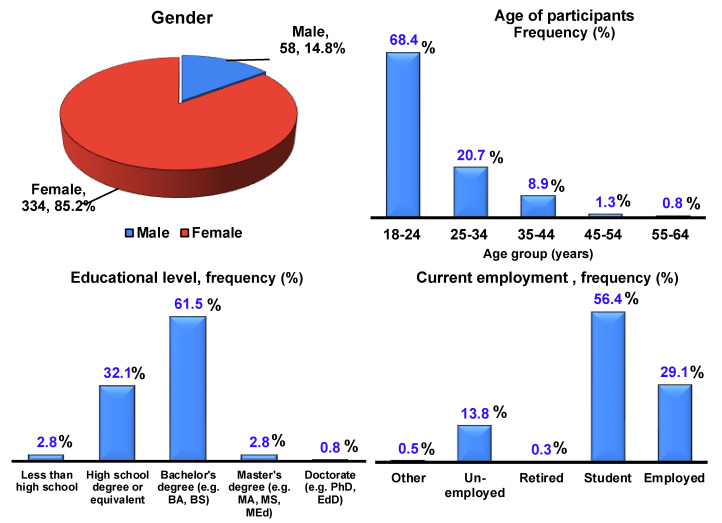
Sociodemographic variables.

Four sociodemographic variables were included; we collected 408 responses to ensure accuracy and excluded individuals under the age of 18 and those residing outside the UAE. A total of 392 respondents qualified based on our inclusion criteria (Figure 1).

The survey data revealed that the majority of participants identified as female (85.2%), with a smaller percentage identifying as male (14.8%). Regarding age distribution, most participants (68.4%) fell into the 18–24 age group, followed by 25–34 years old (20.7%). The representation decreases steadily in older age groups. 

Regarding educational level, most participants reported holding a bachelor’s degree (61.5%), followed by those with a high school degree or equivalent (32.1%). A smaller percentage reported having a master’s degree (2.8%) or a doctorate (0.8%), with only a minority indicating less than a high school education (2.8%). This distribution suggests a generally educated sample with a limited representation of postgraduate degrees. Regarding current employment status, most participants were students (56.4%), followed by those employed (29.1%). Unemployment was reported at 13.8% (Figure 1).

### 3.3. Descriptive Statistics of the Constructs

The respondents were asked to rate their agreement following a five-point Likert scale ranging from 1 to 5.

The descriptive statistics of probiotic and prebiotic knowledge, attitudes, and practices among 392 respondents show that participants generally rated moderate on a five-point Likert scale. The mean (± SD) knowledge scores (*M* = 2.70 ± 1.2) indicate a positive orientation towards probiotics and prebiotics among individuals that have poor knowledge (19.1%), fair knowledge (23.7%), good knowledge (33.7%), very good knowledge (19.4%), and excellent knowledge (4.1%). 

**Figure 2:**
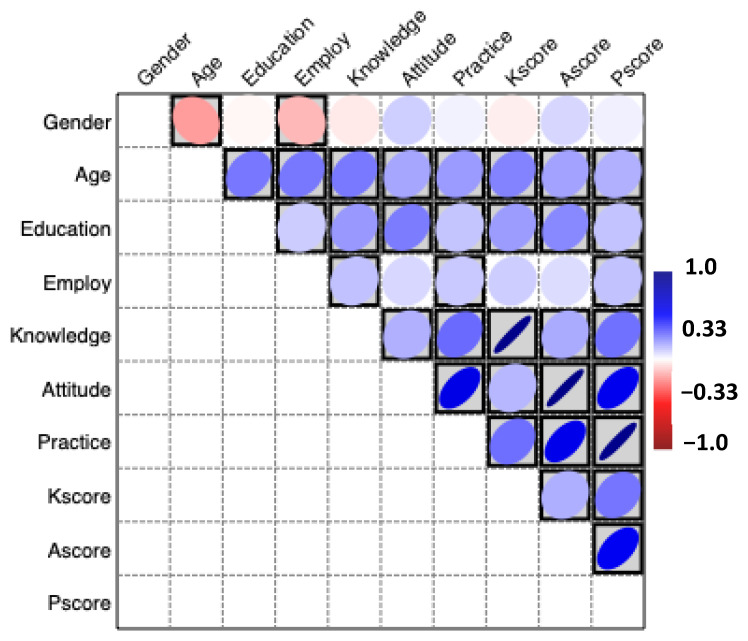
Blue-red heatmap presenting the Pearson’s correlation coefficient for the interrelationships between gender, age, education, employment, and total knowledge, attitude, and practice scores.

The mean (± SD) scores for knowledge variables from KQ1, KQ2, KQ3, KQ4, KQ5, and KQ6 were 2.7 ± 2.0, 2.5 ± 2.0, 2.7 ± 3.0, 2.5 ± 2.0, 3.6 ± 4.0, and 2.3 ± 2.0, respectively. The overall average knowledge scores were 2.7 ± 2.7, with a highly significant (*p* < 0.001) difference between scores as revealed by the Chi-square test (Table 5 and Table 6, Figure 2).

**Figure 3:**
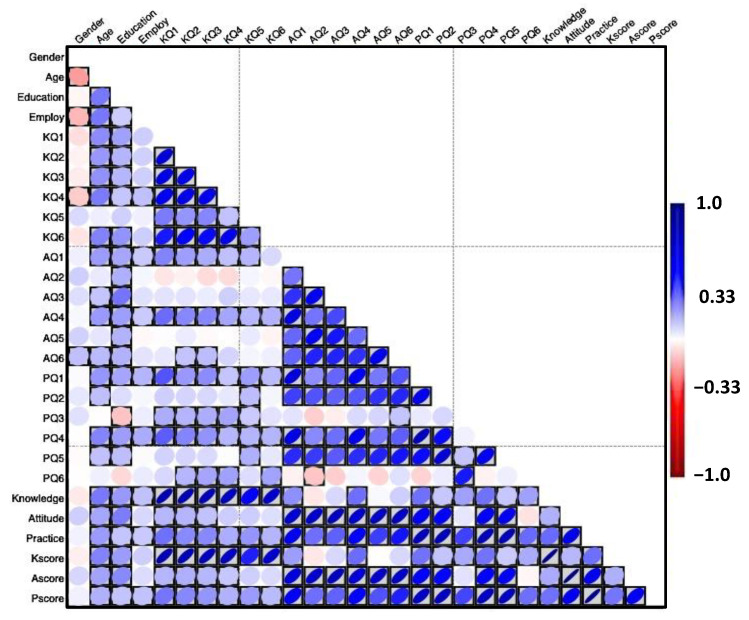
Blue-red heatmap presenting the Pearson’s correlation coefficient for the interrelationships between study variables.

The mean attitude scores were *M* = 3.1 among individuals that have poor attitudes (7.1%), fair attitudes (14.5%), good attitudes (47.2%), very good attitudes (18.6%), and excellent attitudes (12.5%) regarding probiotics and prebiotics. Generally, the mean (± SD) scores for attitude variables from AQ1, AQ2, AQ3, AQ4, AQ5, and AQ6 were 3.5 ± 1.2, 2.8 ± 1.1, 3.0 ± 1.3, 3.4 ± 1.2, 2.9 ± 1.1, and 3.0 ± 1.2, respectively. The overall average attitude scores were 3.1 ± 0.9, with a highly significant (*p* < 0.001) difference between scores as revealed by the Chi-square test (Table 7 and Table 8, Figure 3). 

**Table 6:** Descriptive statistics for probiotic and prebiotic knowledge in terms of frequency (n, %) and the Chi-square test.

Knowledge Questions	No Knowledge		Little Knowledge		Neutral (Undecided)		Medium Knowledge		High Knowledge		Chi-Square
	n	%	n	%	n	%	n	%	n	%	*p*-Value
Knowledge about probiotics?	87	22.2	111	28.3	64	16.3	111	28.3	19	4.8	<0.001 ***
Knowledge about prebiotics?	97	24.7	119	30.4	72	18.4	86	21.9	18	4.6	<0.001 ***
Knowledge of probiotic and prebiotic health benefits?	83	21.2	98	25.0	103	26.3	83	21.2	25	6.4	<0.001 ***
Knowledge of prebiotic types?	98	25.0	115	29.3	95	24.2	67	17.1	17	4.3	<0.001 ***
Questions	None		Chemicals in food		synthetic drugs		Natural plant products		Live microorganisms		Chi-square
	n	%	n	%	n	%	n	%	n	%	*p*-value
What do you think are the constituents of probiotics?	72	18.4	30	7.7	47	12.0	82	20.9	161	41.1	<0.001 ***
Questions	Not aware		Little aware		Neutral		Medium aware		High aware		Chi-square
	n	%	n	%	n	%	n	%	n	%	*p*-value
Are you aware of prebiotics, and how do they differ from probiotics?	108	27.6	125	31.9	86	21.9	62	15.8	11	2.8	<0.001 ***
Knowledge score	75	19.1	93	23.7	132	33.7	76	19.4	16	4.1	<0.001 ***

*** Highly significant at *p* < 0.001.

**Table 7:** Descriptive statistics for probiotic and prebiotic attitudes in terms of mean, median, mode, standard deviation, quartiles, and the Chi-square test.

Attitude Variables (Questions)	Mean	Median	Mode	SD	Percentiles		Chi-Square
					25	75	
Probiotic foods on the table represent health?	3.5	3.0	3.0	1.2	3.0	5.0	<0.001 ***
Probiotics cause decreased absorption of essential minerals?	2.8	3.0	3.0	1.1	2.0	3.0	<0.001 ***
Products containing probiotics are not easily digested?	3.0	3.0	3.0	1.3	2.0	3.0	<0.001 ***
Prebiotic foods on the table represent health?	3.4	3.0	3.0	1.2	3.0	4.8	<0.001 ***
Prebiotics cause decreased absorption of essential minerals?	2.9	3.0	3.0	1.1	2.0	3.0	<0.001 ***
Products containing prebiotics are not easily digested?	3.0	3.0	3.0	1.2	2.0	3.0	<0.001 ***
Attitudes	3.1	3.0	3.0	0.9	2.7	3.7	<0.001 ***
Attitudes score	3.1	3.0	3.0	1.0	3.0	4.0	<0.001 ***

*** Highly significant at *p* < 0.001. SD, standard deviation.

**Table 8:** Descriptive statistics of probiotic and prebiotic attitudes in terms of frequency (n, %) and the Chi-square test.

Attitude Variables (Questions)	Strongly Disagree		Disagree		Neutral (Undecided)		Agree		Strongly Agree		Chi-Square *p*-Value
	n	%	n	%	n	%	n	%	n	%	
Probiotic foods on the table represent health?	34	8.7	30	7.7	134	34.2	80	20.4	114	29.1	<0.001 ***
Probiotics cause decreased absorption of essential minerals?	41	10.5	112	28.6	176	44.9	10	2.6	53	13.5	<0.001 ***
Products containing probiotics are not easily digested?	49	12.5	84	21.4	162	41.3	10	2.6	87	22.2	<0.001 ***
Prebiotic foods on the table represent health?	34	8.7	47	12.0	142	36.2	71	18.1	98	25.0	<0.001 ***
Prebiotics cause decreased absorption of essential minerals?	34	8.7	103	26.3	180	45.9	12	3.1	63	16.1	<0.001 ***
Products containing prebiotics are not easily digested?	49	12.5	77	19.6	175	44.6	12	3.1	79	20.2	<0.001 ***
Attitudes score	28	7.1	57	14.5	185	47.2	73	18.6	49	12.5	<0.001 ***

*** Highly significant at *p* < 0.001.

The mean (± SD) practice scores were *M* = 3.04 ± 0.8 among individuals that have poor practices (5.4%), fair practices (19.6%), good practices (43.1%), very good practices (27.0%), and excellent practices (4.8%) regarding probiotics and prebiotics. The results were all above the midpoint. The standard deviations (knowledge: SD = 0.89; attitudes: SD = 0.85; practices: SD = 0.75) suggest a moderate spread of responses clustered around the mean. 

The range of scores, with the maximum at 5.00 for all constructs and the minimum score lowest for knowledge (2.00), indicates some variability, particularly in the practical application of probiotic and prebiotic knowledge and attitudes. Moreover, the mean (±SD) scores for practice variables from PQ1, PQ2, PQ3, PQ4, PQ5, and PQ6 were 3.6 ± 1.2, 3.2 ± 1.3, 2.5 ± 0.9, 3.4 ± 1.2, 3.1 ± 1.2, and 2.5 ± 0.9, respectively. The overall average practice scores were 3.1 ± 0.9, with a highly significant (*p* < 0.001) difference between scores as revealed by the Chi-square test (Table 9, Figure 3). 

Table 5 and Table 6 present a comprehensive examination of participants’ understanding of probiotics and prebiotics, uncovering notable disparities in levels of awareness. The Chi-square tests conducted on all knowledge questions revealed highly significant differences (*p* < 0.001), indicating a wide range of understanding among participants. For example, 22.2% of participants indicated a lack of familiarity with probiotics, whereas only 4.8% stated a significant level of knowledge. 

Similarly, the data on prebiotics revealed that 24.7% of individuals have no knowledge about prebiotics, while only 4.6% possess a high level of knowledge. The distribution of knowledge regarding the health benefits of probiotics and prebiotics revealed that 21.2% of respondents had no knowledge, 25.0% had limited knowledge, 26.3% were neutral, 21.2% had moderate knowledge, and only 6.4% had extensive knowledge. This distribution revealed a significant disparity in comprehension, as the majority of respondents belong to the lower to mid-level knowledge brackets.

The respondents’ knowledge of prebiotic types varied across different awareness levels: 25.0% had no knowledge, 29.3% had limited knowledge, 24.2% were neutral, 17.1% had moderate knowledge, and only 4.3% were highly knowledgeable. 

Significantly, 41.1% accurately recognized that live microorganisms are components of probiotics, emphasizing an important aspect of knowledge. There was a general lack of awareness regarding the distinctions between prebiotics and probiotics, with only 2.8% of individuals having a high level of awareness. The distribution of knowledge scores was generally biased towards the lower range, as 19.1% of participants exhibited low knowledge, while only 4.1% displayed excellent knowledge. These findings emphasized the need for focused educational programs to improve public comprehension and awareness of probiotics and prebiotics, thus promoting healthier habits and results. 

The Chi-square tests for all sets of questions revealed highly significant differences (*p* < 0.001), indicating that these knowledge gaps are statistically significant and not attributable to random chance. These findings emphasized the urgent requirement for improved educational programs to raise public awareness about the health advantages and different varieties of prebiotics and probiotics, thus encouraging better health results through well-informed dietary decisions.

Table 8 thoroughly examines participants’ viewpoints on probiotics and prebiotics, uncovering notable differences in their perceptions. The Chi-square tests for all attitude questions revealed statistically significant differences (*p* < 0.001), indicating a wide range of participant opinions. When questioned about the relationship between probiotic foods and health, 34.2% of participants had a neutral opinion, while 29.1% strongly agreed, indicating a division between positive and uncertain attitudes. 

In relation to the notion that probiotics lead to reduced absorption of vital minerals, a significant 44.9% of individuals maintained a neutral stance, while 28.6% disagreed and 13.5% strongly agreed, indicating varied opinions regarding the influence of probiotics on mineral absorption. Similarly, 41.3% of participants expressed a neutral opinion regarding the ease of digestion of probiotic products, whereas 22.2% strongly concurred that these products are not easily digested. 

The survey results also indicated that 36.2% of individuals had a neutral attitude towards prebiotic foods, while 25.0% strongly agreed that these foods benefit health. The issue of prebiotics potentially leading to reduced mineral absorption received a 45.9% neutral response and a 16.1% strong agreement, underscoring the existence of varied perspectives. Ultimately, 44.6% of the participants expressed a neutral stance regarding the digestibility of prebiotic products, while 20.2% strongly affirmed that these products are not easily digested. These findings highlighted the necessity for focused educational efforts to address misunderstandings and improve public comprehension of the advantages and characteristics of probiotics and prebiotics, thus promoting better-informed health decisions.

Table 10 thoroughly examined the participants’ practices regarding probiotics and prebiotics, highlighting notable behavioral differences. The Chi-square tests for all practice-related questions revealed statistically significant differences (*p* < 0.001), indicating a wide range of participant practices. When questioned about the safety of probiotics, 29.3% of participants expressed strong agreement, 29.3% had a neutral stance, and 9.2% disagreed, indicating varying levels of confidence in the safety of probiotics. In terms of the efficacy of probiotics in addressing gastrointestinal-related ailments, 42.6% of respondents expressed a neutral stance, while 25.8% strongly agreed, suggesting a moderate level of confidence in their therapeutic advantages.

The consumption patterns of probiotic food products exhibited significant variation, with 44.1% of the participants consuming them infrequently and only 2.0% consuming them consistently, indicating rare usage (Table 10). When assessing the safety of prebiotics, 32.9% of individuals expressed moderate conviction, while 25.5% strongly concurred regarding their safety. A total of 43.6% of respondents expressed a moderate belief in the effectiveness of prebiotics for gastrointestinal issues, while 22.4% strongly believed in their effectiveness. This indicates a positive but cautious attitude towards the benefits of prebiotics. 

Similarly, the analysis of prebiotic food consumption revealed that a mere 2.8% of participants reported consuming them consistently, while 38.8% reported rarely consuming them. This suggests a generally infrequent consumption pattern.

In general, the practice scores exhibited a diverse range of behaviors, with 43.1% of participants displaying moderate levels of practice, while only 4.8% demonstrated excellent practices regarding probiotics and prebiotics. These findings also emphasized the necessity for improved educational initiatives to advocate for more effective and uniform health practices regarding probiotics and prebiotics, with the goal of enhancing public health outcomes by encouraging informed and regular utilization.

### 3.4. Correlation Tests of Probiotics and Prebiotics Knowledge, Attitudes, and Practices

The Pearson correlations among probiotics and prebiotics knowledge, attitudes, and practices for 392 respondents revealed significant relationships (Table 11).

The results indicated a significant correlation between knowledge and age (r = 0.261, *p* < 0.001), suggesting that individuals with higher levels of probiotic and prebiotic knowledge have a significant positive relationship with age (Table 11). Similarly, results also indicated a significant correlation between practice and age (r = 0.198, *p* < 0.001), suggesting that individuals with higher levels of probiotic and prebiotic attitudes also have a significant positive relationship with age (Table 11). 

The results also revealed the highest positive correlation between the highest education and knowledge (r = 0.202, *p* < 0.001), indicating that individuals with the highest education have a significant positive relationship with attitudes towards probiotics and prebiotics (Table 11). These significant correlations at the 0.01 level (two-tailed test) suggest a robust interrelationship among knowledge, attitudes, and practices related to probiotics and prebiotics and sociodemographic variables, highlighting the interconnectedness of these constructs in the context of probiotics and prebiotics (Table 11). 

In Figure 2 and Figure 3, the Pearson coefficient correlation matrix is also presented as a blue–red heatmap. Blue indicates a positive correlation, red indicates a negative inverse relationship, and white indicates no correlation. In addition, the boxed blue or red color indicates a significant correlation, as revealed by the two-tailed test. 

The results indicated a significant direct correlation between knowledge and practices (r = 0.291, *p* < 0.001), suggesting that individuals with higher levels of probiotic and prebiotic knowledge are more likely to adopt sustainable practices. The results also revealed a positive correlation between attitudes and practices (r = 0.593, *p* < 0.001), indicating that individuals with positive attitudes towards probiotics and prebiotics tend to engage in more sustainable practices. 

However, no significant correlation was observed between knowledge and attitudes (r = 0.154, *p* = 0.002), suggesting that possessing knowledge alone may not necessarily lead to positive attitudes towards probiotics and prebiotics. These significant correlations at the 0.01 level (two-tailed test) suggest a robust interrelationship among knowledge, attitudes, and practices related to probiotics and prebiotics, highlighting the interconnectedness of these constructs in the context of probiotics and prebiotics (Table 12).

### 3.5. Regression of Probiotics and Prebiotics Practice on Attitudes and Knowledge

The regression analysis demonstrates a significant and impactful relationship between knowledge and attitudes towards probiotic and prebiotic practices. The results highlighted the importance of knowledge and attitudes in influencing sustainable practices, with attitudes having a slightly more pronounced effect.

In the model summary (Table 13), the predictors, knowledge and attitudes, showed a moderate correlation with practices (R = 0.627), indicating a substantial relationship. The R square value of 0.393 suggests that the model, in substantial proportion, explains 39.3% of the variance in practices. The adjusted R square, at 0.390, adjusts this value slightly for the number of predictors but still indicates significant explanatory power. The Durbin–Watson statistic of 1.952 points to a moderate level of autocorrelation in the residuals, which is within acceptable limits and does not detract from the model’s validity.

The ANOVA results further confirmed the model’s effectiveness. The regression model was statistically significant (F(2, 389) = 125.988, *p* < 0.001), demonstrating a strong relationship between the independent variables (knowledge and attitudes) and the dependent variable (practices). The division of total variance into regression (86.801) and residual (134.004) parts underlines the model’s capacity to account for significant variability in practices.

The coefficients table (Table 14) provides detailed insights into the individual predictors. Both knowledge and attitudes are significant predictors of practices, with knowledge having a positive effect (B = 0.172, t = 5.118, *p* < 0.001) and a moderate influence (standardized beta = 0.205). Attitudes showed a stronger positive effect on practices (B = 0.491, t = 14.058, *p* < 0.001), with a higher beta value of 0.562, indicating a more substantial impact on practices. The collinearity statistics for both predictors are within acceptable ranges, suggesting that multicollinearity is not a concern in this model.

## 4. Discussion

The present study aimed to evaluate the level of KAP regarding probiotics and prebiotics among the population of the UAE. These elements, which signify distinct aspects of behaviour, are crucial for comprehending how individuals perceive and respond to health-related matters.

This study’s findings suggest that a significant proportion (33.7%) of the respondents have substantial knowledge about probiotics. In a different study, the researchers investigated the KAP of health science undergraduates in relation to probiotics for the gut–skin axis [46]. The findings revealed that 48.0% of the participants displayed satisfactory knowledge. In a study conducted in Oman by AbuKhader et al. [54] on probiotics in medical sciences education, it was found that both medicine students (MD) (89.3%) and dental students (BDS) (95%) believe that they do not have enough knowledge about probiotics, which was statistically significant (*p* < 0.028) [54]. In this study, the distribution of knowledge scores was generally biased towards the lower range, as 19.1% of participants exhibited medium knowledge, while only 4.1% displayed excellent knowledge. However, in another study by Fijan et al. [55] about health professionals’ knowledge of probiotics, most of the respondents evaluated their knowledge of probiotics as medium (36.4%) or good (36.2%), with only 8.9% of the respondents rating it as excellent [55]. 

A separate study by Barqawi et al. [48] investigated the KAP of the population in the UAE regarding the microbiota and its composition. The findings revealed a limited understanding of the microbiota, with only 29.3% of the population demonstrating good knowledge. Notably, university students or healthcare professionals were found to have a higher level of knowledge about the microbiota [48]. Although probiotics are known, only 9.1% of individuals demonstrated a high level of understanding. The study found that sociodemographic factors did not correlate significantly with KAP regarding probiotics [48]. 

In another study conducted by Fijan et al. [55], no statistical difference was found in knowledge between male and female health workers. Moreover, a study that investigated consumers’ knowledge levels and consumption status of probiotics and prebiotic products found that there were statistically significant differences in knowledge of probiotics and prebiotics by sex (*p* < 0.001 and *p* < 0.001, respectively) [56]. 

Meanwhile, a study conducted in Romania by Precup et al. [24] examined consumer awareness, knowledge, and interest in prebiotics, uncovering disparities in knowledge levels among respondents [24]. A strong correlation was observed between age and knowledge levels, indicating that younger respondents exhibited greater awareness and comprehension of prebiotics compared to older respondents (*p* < 0.05) [24]. A total of 33.7% of the respondents possessed substantial knowledge about probiotics. Similarly, in Precup et al. [24] study, the results indicated a significant correlation between knowledge and age (r = 0.261, *p* < 0.001), suggesting that individuals with higher levels of knowledge about probiotics and prebiotics have a significant positive relationship with age. In a similar way, the results of Precup et al. [24] also revealed the highest positive correlation between the highest education and knowledge (r = 0.202, *p* < 0.001), indicating that individuals with the highest education have more significant positive attitudes towards probiotics and prebiotics [24]. 

Furthermore, a separate investigation conducted in Riyadh, Saudi Arabia, by Al hossan et al. [57] examined the KAP of probiotics among healthcare students [57]. The study revealed a significant disparity in the age groups of students, with those above 25 years old exhibiting the highest average score (*p* = 0.001) [57]. Conversely, this study’s findings suggest a favourable disposition (3.1 ± 0.85) towards probiotics and prebiotics. 

Another study [58] investigated the relationship between KAP and probiotics for the digestive system among health science students and revealed that 52.9% of the participants held a favourable attitude toward probiotics. In a study conducted on knowledge and attitude towards probiotics, a positive, weak correlation was found between knowledge and attitude [58]. However, Babina et al. [58] study showed no significant correlation between knowledge and attitudes (r = 0.154, *p* = 0.002), suggesting that possessing knowledge alone may not necessarily lead to positive attitudes towards probiotics and prebiotics. Likewise, a different study showed no significant correlation between knowledge and attitude [7].

In conclusion, the newly reported studies on probiotics and prebiotics have positive implications for clinical practice and public health interventions. Medical practitioners can use this information to develop treatment plans for conditions such as mood disorders, cognitive decline, and IBS. Integrating probiotics and prebiotics into comprehensive care programs can assist doctors in treating fundamental dysfunctions of the gut-brain axis in a more comprehensive manner. In addition, promoting the incorporation of foods rich in probiotics and prebiotic fibers in dietary recommendations could aid public health efforts by empowering individuals to take proactive steps towards enhancing their overall health and well-being.

The significance of KAP research pertaining to prebiotics and probiotics is of utmost importance, particularly within the context of the UAE. Gaining a comprehensive understanding of the population’s KAP is crucial for obtaining valuable insights into how these agents that promote health are perceived and used. This research emphasizes the specific areas where educational interventions are necessary and identifies the lack of awareness that can be addressed through public health campaigns. Furthermore, KAP studies can provide valuable insights to policymakers and healthcare providers regarding the most efficient approaches to promote the acceptance and incorporation of probiotics and prebiotics into everyday health routines.

Conducting KAP research specific to the UAE’s cultural and dietary practices is crucial in creating customized health promotion strategies that effectively connect with the population. This has the potential to enhance public health outcomes and significantly enhance community health overall. To promote a knowledgeable and health-conscious society, it is imperative for the UAE to integrate the findings from KAP research on probiotics and prebiotics into its healthcare initiatives.

Further investigation is also warranted to examine the enduring impacts of probiotics and prebiotics on different health conditions, specifically in diverse demographic groups within the UAE.

Implementing educational programs targeting the public and healthcare professionals can enhance knowledge and attitudes regarding probiotics and prebiotics, ultimately improving health outcomes.

## Figures and Tables

**Table 4:** Reliability analysis.

ID	Scales	Number of Items	Cronbach Alpha Reliability
1	Knowledge	06	0.809
2	Attitudes	06	0.808
3	Practices	06	0.728
Total		18	0.840

**Table 5:** Descriptive statistics for probiotic and prebiotic knowledge in terms of mean, median, mode, standard deviation, quartiles, and the Chi-square test.

Knowledge Variables (Questions)	Mean	Median	Mode	SD	Quartiles		Chi-Square
					25	75	
How is your knowledge about probiotics?	2.7	2.0	2.0	1.2	2.0	4.0	<0.001 ***
How is your knowledge about prebiotics?	2.5	2.0	2.0	1.2	2.0	4.0	<0.001 ***
Knowledge of probiotic and prebiotic health benefits?	2.7	3.0	3.0	1.2	2.0	4.0	<0.001 ***
Knowledge of prebiotic types?	2.5	2.0	2.0	1.2	1.3	3.0	<0.001 ***
What do you think are the constituents of probiotics?	3.6	4.0	5.0	1.5	2.0	5.0	<0.001 ***
Are you aware of prebiotics, and how do they differ from probiotics?	2.3	2.0	2.0	1.1	1.0	3.0	<0.001 ***
Knowledge	2.7	0.89	3.5	0.9	2.2	3.3	<0.001 ***
Knowledge score	2.7	3.0	3.0	1.1	2.0	3.0	<0.001 ***

*** Highly significant at *p* < 0.001. SD, standard deviation.

**Table 9:** Descriptive statistics for probiotic and prebiotic practices in terms of mean, median, mode, standard deviation, quartiles, and the Chi-square test.

Variables (Questions)	Mean	Median	Mode	SD	Quartiles		Chi-Square
					25	75	
I believe that probiotics are safe.	3.6	4.0	3.0	1.2	3.0	5.0	<0.001 ***
I believe that probiotics are effective in the treatment of certain gastrointestinal-related illnesses or symptoms.	3.2	3.0	3.0	1.3	2.0	5.0	<0.001 ***
Do you consume food products with probiotics?	2.5	2.0	2.0	0.9	2.0	3.0	<0.001 ***
I believe that prebiotics are safe.	3.4	3.0	3.0	1.2	3.0	5.0	<0.001 ***
I believe that prebiotics are effective in the treatment of certain gastrointestinal-related illnesses or symptoms.	3.1	3.0	3.0	1.2	2.0	4.0	<0.001 ***
Do you consume food products with prebiotics?	2.5	2.0	2.0	0.9	2.0	3.0	<0.001 ***
Practices	3.0	3.0	3.0	0.8	2.5	3.5	<0.001 ***
Practice score	3.1	3.0	3.0	0.9	2.3	4.0	<0.001 ***

*** Highly significant at *p* < 0.001. SD, standard deviation.

**Table 10:** Descriptive statistics of probiotic and prebiotic practices in terms of frequency (n, %) and the Chi-square test.

Questions	Strongly Disagree		Disagree		Neutral (Undecided)		Agree		Strongly Agree		Chi-Square
	n	%	n	%	n	%	n	%	n	%	*p*-Value
Probiotics are safe.	34	8.7	36	9.2	115	29.3	92	23.5	115	29.3	<0.001 ***
I Believe that probiotics are effective in the treatment of certain gastrointestinal-related illnesses or symptoms.	39	9.9	67	17.1	167	42.6	18	4.6	101	25.8	<0.001 ***
Questions	Never		Rarely		Occasionally		Frequently		Always		
Do you consume food products with probiotics?	48	12.2	173	44.1	116	29.6	47	12.0	8	2.0	<0.001 ***
	Not at all		Slightly		Moderately		Very much		Extremely		
I believe that prebiotics are safe.	40	10.2	35	8.9	129	32.9	88	22.4	100	25.5	<0.001 ***
I believe that prebiotics are effective in the treatment of certain gastrointestinal-related illnesses or symptoms.	42	10.7	72	18.4	171	43.6	19	4.8	88	22.4	<0.001 ***
Do you consume food products with prebiotics?	53	13.5	152	38.8	138	35.2	38	9.7	11	2.8	<0.001 ***
Practice score	21	5.4	77	19.6	169	43.1	106	27.0	19	4.8	<0.001 ***

*** Highly significant at *p* < 0.001.

**Table 11:** Correlations between probiotic and prebiotic knowledge, attitudes, and practices among sociodemographic variables.

		Age	Gender	Education	Employment
Knowledge	r	0.261	−0.041	0.202	0.120
	*p*-value	<0.001 ***	0.415	<0.001 ***	0.017 *
Attitudes	r	0.174	0.096	0.256	0.078
	*p*-value	<0.001 ***	0.058	<0.001 ***	0.123
Practices	r	0.198	0.024	0.115	0.106
	*p*-value	<0.001 ***	0.638	0.022 *	0.036 *

* Significant at *p* < 0.05; *** highly significant at *p* < 0.001.

**Table 12:** Correlations between probiotic and prebiotic knowledge, attitudes, and practices.

		Knowledge	Attitudes	Practices
Attitudes	r	0.154	--	
	*p*-value	0.002 **		
Practices	r	0.0291	0.593	--
	*p*-value	<0.001 ***	<0.001 ***	

** Significant at the 0.01 level (two-tailed test); *** highly significant at *p* < 0.001.

**Table 13:** Probiotics and prebiotics practice model summary with attitudes and knowledge as predictors and practice as a dependent variable.

R	R Square	Adjusted R Square	SE of Estimate	Change Statistics					Durbin–Watson
				R Square Change	F-Change	DF1	DF2	Significant F Change	
0.627 ^a^	0.393	0.390	0.58693	0.39	125.988	2	389	<0.001 ***	1.952

*** Highly significant at *p* < 0.001. ^a^ Attitudes and knowledge as predictors and practice as a dependent variable. SE, standard error; DF, degree of freedom.

**Table 14:** Probiotic and prebiotic practice coefficients using practice as a dependent variable.

Model		Unstandardized Coefficients		Standardized Coefficients	t	Significance	Collinearity Statistics	
		B	SE	Beta			Tolerance	VIF
1	(Constant)	1.053	0.134		7.9	<0.001 ***		
	Knowledge	0.172	0.034	0.205	5.1	<0.001 ***	0.976	1.024
	Attitudes	0.491	0.035	0.562	14.1	<0.001 ***	0.976	1.024

*** Highly significant at *p* < 0.001. B, regression coefficients; SE, standard error; VIF, variance inflation factor.

## Data Availability

The original contributions presented in the study are included in the article, further inquiries can be directed to the corresponding author.
